# Utilizing anomalous signals for element identification in macromolecular crystallography

**DOI:** 10.1107/S2059798324008659

**Published:** 2024-09-18

**Authors:** Kamel El Omari, Ismay Forsyth, Ramona Duman, Christian M. Orr, Vitaliy Mykhaylyk, Erika J. Mancini, Armin Wagner

**Affiliations:** a Diamond Light Source Harwell Science and Innovation Campus DidcotOX11 0DE United Kingdom; b Research Complex at Harwell Rutherford Appleton Laboratory DidcotOX11 0FA United Kingdom; c School of Life Sciences University of Sussex Falmer BrightonBN1 9QG United Kingdom; Deutsches Elektronen-Synchrotron, Germany

**Keywords:** crystallography, anomalous scattering, element identification

## Abstract

*AlphaFold*2 has revolutionized structural biology by offering unparalleled accuracy in predicting protein structures. Traditional methods for determining protein structures, such as X-ray crystallography and cryo-electron microscopy, are often time-consuming and resource-intensive. *AlphaFold*2 provides models that are valuable for molecular replacement, aiding in model building and docking into electron density or potential maps. However, despite its capabilities, models from *AlphaFold*2 do not consistently match the accuracy of experimentally determined structures, need to be validated experimentally and currently miss some crucial information, such as post-translational modifications, ligands and bound ions. In this paper, the advantages are explored of collecting X-ray anomalous data to identify chemical elements, such as metal ions, which are key to understanding certain structures and functions of proteins. This is achieved through methods such as calculating anomalous difference Fourier maps or refining the imaginary component of the anomalous scattering factor *f*′′. Anomalous data can serve as a valuable complement to the information provided by *AlphaFold*2 models and this is particularly significant in elucidating the roles of metal ions.

## Abstract 2

This article examines the application of anomalous scattering for the identification of elements within crystal structures of macromolecules.

## 1. Introduction

The field of structural biology underwent a significant transformation in 2020 following the introduction of *AlphaFold*2 (AF2; Jumper *et al.*, 2021 ▸), which revolutionized protein structure prediction. Structural biologists swiftly embraced the benefits of AF2 models (Perrakis & Sixma, 2021 ▸), such as guiding construct design, predicting protein interactions and largely decreasing the need for experimental phasing in structure determination through the use of molecular replacement in crystallography (Millán *et al.*, 2021 ▸) and model docking in electron cryo-electron microscopy. In numerous instances, AF2 predictions aligned with experimental maps. However, in some cases even predictions with very high confidence exhibited disparities with experimental maps (Thornton *et al.*, 2021 ▸). It has been suggested to prioritize the consideration of the confidence level of predictions when interpreting AF2 results and to advocate for experimental structure determination to validate structural details (Terwilliger *et al.*, 2024 ▸).

Experimental structure determination not only enables the validation of AF2 models but also incorporates anomalous scattering information which can improve the quality of the models. Anomalous data can provide additional insights into both the structure and the functionality of the protein. This can be achieved by providing unbiased electron-density maps, confirming the positions of S atoms or identifying other elements. Initially, anomalous scattering was primarily utilized for experimental phasing, but it can also be employed for the identification of anomalous scatterers. Important biological elements such as P and S atoms or metal-ion cofactors can be experimentally identified in crystal structures. Presently, AF2 predictions do not include coordinates for metal ions. However, algorithms such as *AlphaFill* attempt to address this limitation (Hekkelman *et al.*, 2023 ▸). Unfortunately, the assignments of such ions in the Protein Data Bank (PDB; wwPDBconsortium, 2019 ▸) are not always experimentally validated, so training for machine-learning algorithms might be limited to some well characterized and over-represented ions such as zinc.

Normal scattering, in which excited electrons emit X-rays in all directions without phase shift, is an idealized concept. Initially approximated by the classical theory for elastic scattering of free electrons by Thomson (1906 ▸), it crucially assumes free electrons. Consequently, the atomic scattering factor (*f*) for each atom would be directly proportional to the atomic number (*Z*), implying that all atoms scatter X-rays similarly, thereby adhering to Friedel’s law (|*F*_*hkl*_| = |*F*_−*h*−*k*−*l*_|). In reality, electrons are not free but are tightly bound to the nucleus and exhibit resonance at specific wavelengths. When incident X-rays have wavelengths near the absorption edge of an element, anomalous scattering replaces normal scattering: photons are absorbed, causing electron resonance. This process leads to either fluorescence or immediate re-emission of radiation with a 90° phase shift, which is typically implied by anomalous scattering. In this situation, additional components [the real dispersive component (*f*′) and the imaginary absorptive component (*f*′′)] need to be added to the description of the atomic scattering factor: *f*(λ) = *f*_o_ + *f*′(λ) + *if*′′(λ) (reviewed in Liu & Hendrickson, 2017 ▸). When this adjustment occurs, Friedel’s law is broken, resulting in asymmetry between symmetry-related reflections within the same data set, as the *f*′′ phase shift introduces intensity differences called anomalous differences. These anomalous differences are only attributed to the anomalous scatterers present in the crystal; therefore, they can be used for substructure determination or for element identification.

Anomalous scattering can be used to identify or validate elements with absorption edges within the range of wavelengths accessible by synchrotron beamlines (Einsle *et al.*, 2007 ▸; Liu *et al.*, 2013 ▸); some beamlines such as I23 at Diamond Light Source (Wagner *et al.*, 2016 ▸) can extend this range to longer wavelengths and even reach the P *K* edge (λ = 5.76 Å; El Omari *et al.*, 2023 ▸). Absorption edges can be expressed in terms of energies or wavelengths, and these two units are inversely proportional (*E* = *hc*/λ). While wavelengths are used for data collection, energy is more often used to describe absorption edges. In the manuscript, the terms ‘above’ and ‘below’ the absorption edge refer to higher and lower energies of data collection, respectively, and thus shorter and longer wavelengths. Data collections above and below the absorption edge provide sufficient information to identify a chemical element. Should data collection around the absorption edge prove unfeasible, an alternative method known as *f*′′ refinement can aid in element assignment. This has already been implemented in the ion-identification tool in *Phenix* (Echols *et al.*, 2014 ▸). This method entails refining the imaginary component of the anomalous scattering factor *f*′′ for a particular element during the refinement process of the protein structure. The refined values are then compared with the theoretical values at the wavelength of data collection.

The insights gained from collecting anomalous data can play a crucial role in addressing biological questions, particularly in the case of metalloenzymes, where the metal ion may be pivotal to the structure and/or function of the protein. Anomalous data can also be used to determine metal-ion oxidation states using spatially resolved anomalous dispersion (SpReAD; Lennartz *et al.*, 2022 ▸; Spatzal *et al.*, 2016 ▸). Additionally, anomalous data can aid in ligand identification, for example in fragment-based drug design (Ma *et al.*, 2024 ▸).

## 2. Materials and methods

### 2.1. Protein crystallization and structure determination

LMO4, a human transcription factor containing four zinc fingers, was expressed as a construct consisting of the tandem LIM domains of LMO4 (residues 16–152, including C52S/C64S mutations) fused to LDB1LID (residues 336–375) (Deane *et al.*, 2003 ▸). The pET-47b(+) vector was transformed into *Escherichia coli* Rosetta (DE3) pLysS cells, protein expression was induced by the addition of 0.5 m*M* isopropyl β-d-1-thiogalactopyranoside (IPTG) and growth was continued for 18 h at 303 K. The cells were harvested by centrifugation and resuspended in 50 m*M* phosphate buffer pH 7.4, 500 m*M* NaCl, 10 m*M* imidazole, 0.5 m*M* TCEP. The cells were then disrupted by sonication on ice and the lysate was clarified by centrifugation. The supernatant was applied onto an Ni^2+^-charged chelating column equilibrated with lysis buffer. The protein was eluted with a gradient of imidazole. Fractions containing LMO4 were pooled for additional purification using a Superdex 75 gel-filtration column (GE Healthcare) with 300 m*M* NaCl, 50 m*M* Tris pH 7.4, 0.5 m*M* TCEP. Fractions containing LMO4 were pooled and concentrated to 17 mg ml^−1^ using a 10 kDa filter. The best diffracting crystals grew within two days of setup in 0.25 *M* sodium malonate pH 7, 20%(*v*/*w*) PEG 3350. Crystals were cryoprotected with 25%(*v*/*v*) glycerol.

All data collections took place on beamline I23 at Diamond Light Source (DLS), Didcot, United Kingdom (El Omari *et al.*, 2023 ▸; Wagner *et al.*, 2016 ▸) at a temperature of 80 K, with a typical dose for 360° of data being less than 1.5 MGy. LMO4 data sets were collected at three wavelengths (λ = 1.2853, 1.2874 and 1.3051 Å). For each data set, 360° of data were collected with a transmission of 50%, an exposure of 0.1 s, an oscillation of 0.1°, a beam size of 200 × 350 µm and a flux of 3 × 10^8^ photons s^−1^.

Thermolysin from *Bacillus thermoproteolyticus*, purchased from Merck (catalogue No. P1512) as a lyophilized powder, was dissolved to a concentration of 50 mg ml^−1^ in 50 m*M* MES pH 6.0, 45% DMSO, 50 m*M* NaCl. Rod-shaped thermolysin crystals appeared within one to two days in 1.2 *M* ammonium sulfate. These crystals were subsequently soaked in reservoir solution containing 2 m*M* CaCl_2_ without further cryoprotection. Two data sets corresponding to above and below the Ca *K* edge (λ = 3.0685 and 3.0803 Å, respectively) were collected using the interleaved method; 360° of data were collected at each wavelength using 90° wedges. Data sets were collected with an exposure of 0.1 s, an oscillation of 0.1° and a beam size of 110 × 250 µm using a flux in the range 1–5 × 10^10^ photons s^−1^.

Hen egg-white lysozyme, purchased from Sigma (catalogue No. 62971) as a lyophilized powder, was dissolved to a concentration of 10 mg ml^−1^ in 10 m*M* sodium acetate pH 3.8 and crystallized within a day in 100 m*M* sodium acetate pH 4.6, 1 *M* NaCl, 25% ethylene glycol. The crystals did not need further cryoprotection. Three 360° data sets were collected from a laser-shaped lysozyme crystal using the interleaved method (90° sweeps) at wavelengths of 4.1328, 4.5920 and 5.1660 Å, corresponding to above and below the Cl and S *K* edges, respectively. The beam size was adjusted to the size of the crystal (200 × 200 µm) and data sets were recorded with an exposure of 0.1 s and an oscillation of 0.1° using a flux of 3 × 10^10^ photons s^−1^.

The expression, purification, crystallization and structure determination of NaK2K from *Bacillus cereus* have previously been reported (Langan *et al.*, 2018 ▸). The structure and structure factors deposited as PDB entry 6dz1 were used for *f* ′′ refinement.

All data sets were processed with *xia*2 *DIALS* (Winter, 2010 ▸; Winter *et al.*, 2022 ▸) and molecular replacement was automatically carried out with *Phaser* (McCoy *et al.*, 2007 ▸) as implemented in the *DIMPLE* pipeline. PDB entries 2lyz (Diamond, 1974 ▸), 3tmn (Holden & Matthews, 1988 ▸) and 1rut (Deane *et al.*, 2004 ▸) were used as molecular-replacement search models for lysozyme, thermolysin and LMO4, respectively. Refinement was carried out with either *REFMAC*5 (Murshudov *et al.*, 2011 ▸) or *phenix.refine*. Data-collection and refinement statistics are provided in Tables 1 ▸ and 2 ▸, respectively.

### 2.2. Anomalous difference Fourier maps

Anomalous difference Fourier maps and anomalous peak heights were calculated with *ANODE* (Thorn & Sheldrick, 2011 ▸) using the molecular-replacement solution from *DIMPLE* and the *DIALS* reflection file processed by *SHELXC* (Sheldrick, 2010 ▸). Anomalous peak heights are reported in Table 3 ▸.

### 2.3. *f*′′ refinement

The protocol was derived from previously reported studies (Karasawa *et al.*, 2023 ▸; Liu *et al.*, 2013 ▸). Following the completion of the standard structure-refinement process, the Friedel pairs were merged and the *B* factors of the ions were checked against neighbouring contacting atoms. Large differences in *B* factors would indicate a problem with the identity or the occupancy of the element. In the examples reported in this paper the sites were fully occupied, so their occupancy was not refined and was fixed at 1. The last step was solely dedicated to refining *f*′′ (all other parameters such as *B* factors were kept fixed) using *phenix.refine* (Liebschner *et al.*, 2019 ▸), but this time the Friedel pairs were kept separated. If the site is fully occupied, *f*′′ can be refined as a parameter; if the site is not fully occupied, *f*′′ values can be scanned against different occupancy as reported by Karasawa *et al.* (2023 ▸). The results of this refinement were then compared either with experimentally measured *f*′′ values from an X-ray absorption edge scan measured in fluorescence mode or with theoretical values at the wavelength of data collection (Cromer & Liberman, 1981 ▸; Kissel & Pratt, 1990 ▸; Table 4 ▸).

## 3. Results and discussion

X-ray diffraction experimental data can provide insights into the identity and location of anomalous scatterers, particularly if the imaginary component *f*′′ is significant at the data-collection wavelength. In cases where the anomalous signal is weak, increasing the data multiplicity can enhance the signal (Liu *et al.*, 2012 ▸), provided that the radiation damage is within acceptable limits: typically less than 5 MGy for selenomethionine-containing crystals (Holton, 2007 ▸). While anomalous data are commonly associated with phasing, they also prove to be invaluable for element identification. Important biological elements such as manganese, iron, copper and zinc can be identified using most beamlines (wavelengths of ∼0.7–2 Å), while others such as calcium, potassium, chlorine, sulfur and phosphorus can only be identified on long-wavelength beamlines such as I23 at Diamond Light Source (wavelengths up to 5.5 Å; Wagner *et al.*, 2016 ▸).

**Figure 1:**
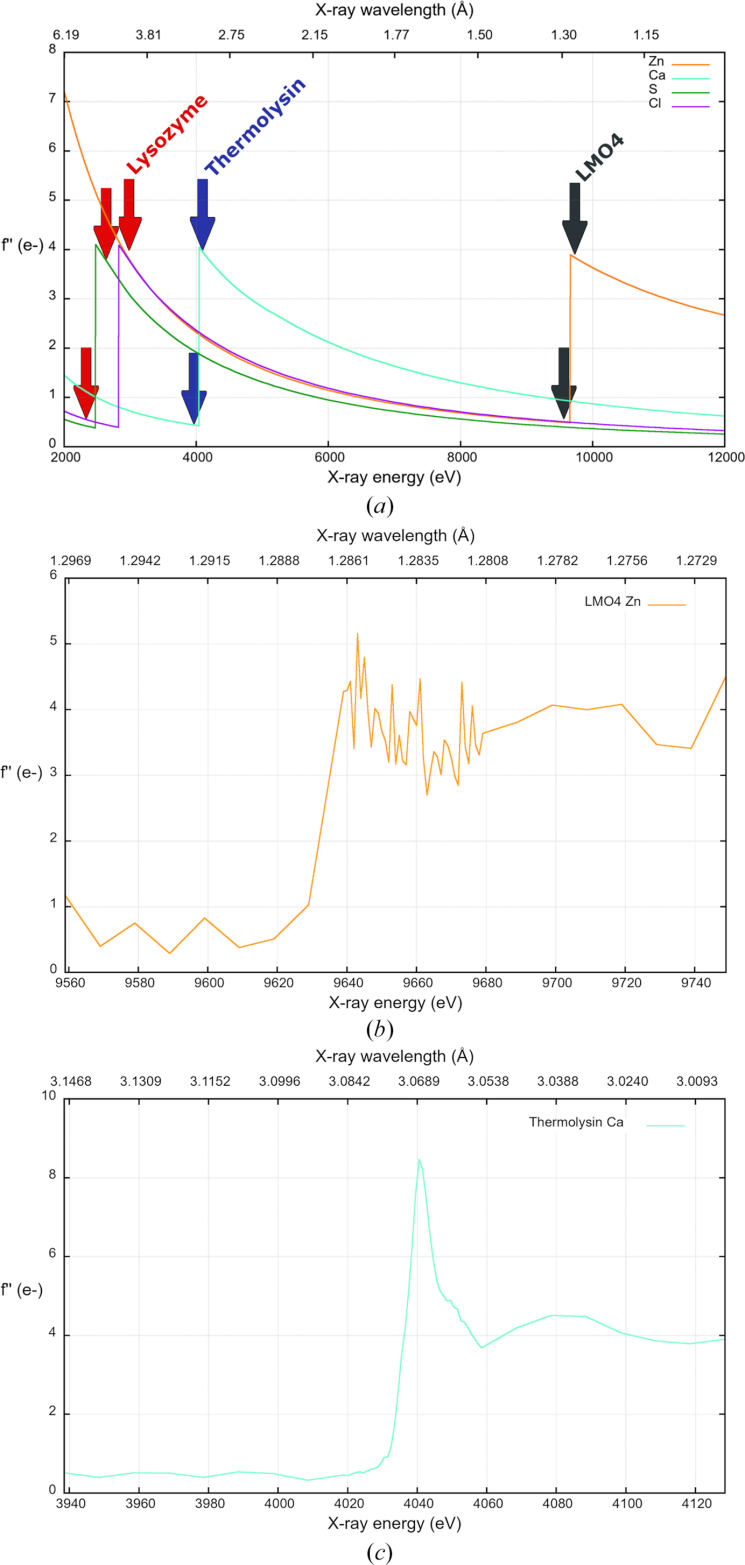
Variation of *f*′′ with X-ray wavelength (or energy) showing absorption *K* edges for sulfur (green), chlorine (magenta), calcium (cyan) and zinc (orange). (*a*) Theoretical *f*′′ values obtained from the website https://skuld.bmsc.washington.edu/scatter/. Wavelengths used for data collections are marked with red, blue and grey arrows for lysozyme, thermolysin and LMO4, respectively. (*b*) Zn *K*-edge absorption-edge scan as determined by *CHOOCH* (Evans & Pettifer, 2001 ▸) measured from an LMO4 crystal. (*c*) Ca *K*-edge absorption edge scan as determined by *CHOOCH* and measured from a thermolysin crystal.

For data collections specifically aimed at element identification, the protocol involves collecting data sets above and below the absorption edge of the target element (Fig. 1 ▸*a*). Indeed, each chemical element has a unique set of absorption edges corresponding to the wavelengths (energies) required to excite electrons in that element to higher energy levels. Above the edge, both the measured anomalous signal and *f*′′ are typically high (*f*′′ = 4 e^−^ at the absorption *K* edges), whereas below the edge the anomalous signal and *f*′′ are either negligible or significantly reduced. By comparing anomalous peak heights or anomalous difference Fourier maps, specific elements can be identified and placed in the crystal structure. This method has successfully been utilized to assign various elements such as potassium (Langan *et al.*, 2018 ▸; Rozov *et al.*, 2019 ▸), calcium (Herdman *et al.*, 2022 ▸) and chlorine (Chukhutsina *et al.*, 2022 ▸).

When only a single wavelength is available, and it may have been collected far from an absorption edge, it is still feasible to assign an element and refine the *f*′′ component of its atomic scattering factor (Liu *et al.*, 2013 ▸). The requirements are that anomalous data are collected, ideally covering 360° to record Friedel pairs, and that *f*′′ is not zero at the wavelength of data collection (*f*′′ values as low as 0.3 e^−^ have been reported; Karasawa *et al.*, 2023 ▸). The refined *f*′′ value can be compared with the theoretical value at a specific wavelength and the identity of the element validated.

### 3.1. Element identification with anomalous difference Fourier maps

Anomalous difference Fourier maps are a type of electron-density map used in X-ray crystallography to visualize the distribution of anomalous scattering. In the program *ANODE*, instead of adding a phase shift to the heavy-atom phases to obtain a starting value for the native protein phase, the phase shift is subtracted from the native phase to obtain the anomalous substructure phase (Thorn & Sheldrick, 2011 ▸). Prior phase information, frequently derived from molecular replacement, is essential to generate these maps. Anomalous difference Fourier map calculations compute the positions of anomalous peaks measured in σ: positive/strong peaks typically indicate regions where the anomalous scatterers are situated, while negative/weak peaks denote areas where they are either absent or less prevalent. In this paper we used this method on three test crystals, LMO4, thermolysin and lysosyme, to identify zinc, calcium and chloride ions, respectively.

Ideally, an X-ray absorption-edge scan should be measured to determine the wavelength at which *f*′′ is maximized (the peak wavelength). Subsequently, two data sets can be acquired, one above and one below the peak. If an X-ray absorption-edge scan is unavailable, the theoretical wavelength for the peak can be utilized instead (Figs. 1 ▸*b* and 1 ▸*c*). However, due to the influence of the chemical environment on the anomalous scatterer, a slight shift may occur. Therefore, it is advisable to collect data a few tenths or hundredths of ångströms away from the theoretical peak. It is crucial to gather data sets with complete anomalous data, typically requiring 360° of data, except in cases of low-symmetry space groups, for which more data might be required and a multi-axis goniometer might be used. Data multiplicity is not as crucial as in SAD phasing, since the phases used to calculate the anomalous difference Fourier maps to locate the anomalous scatterers are obtained from existing refined models. In contrast, in SAD phasing multiplicity is used to enhance the anomalous signal to directly locate the anomalous scatterers as part of the initial structure determination.

The data collection can be interleaved between the two wavelengths, as reported here for the thermolysin and lysozyme data sets, to evenly distribute the radiation damage and ensure that the anomalous differences between data sets are comparable.

**Figure 2:**
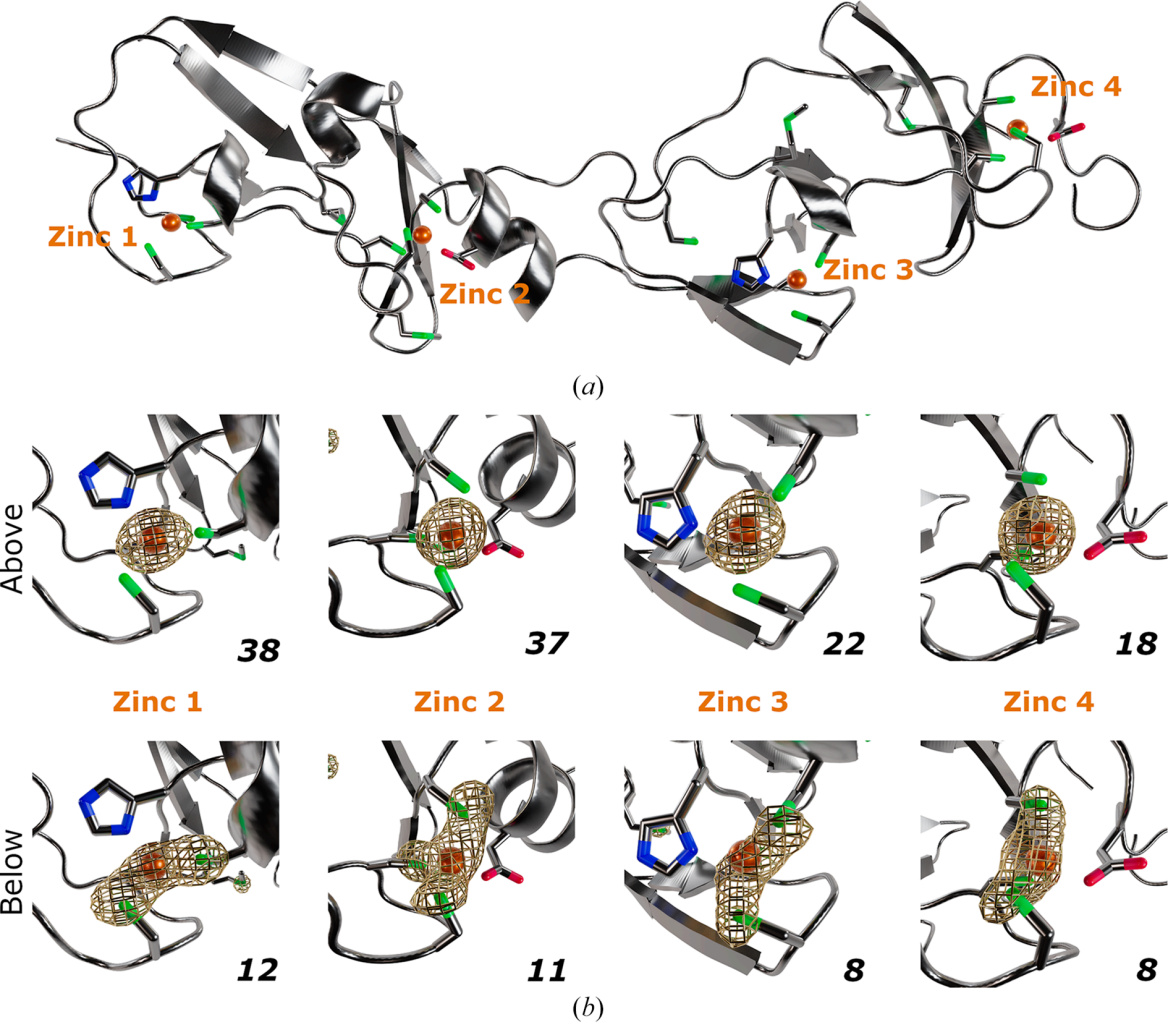
Zinc identification in the LMO4 structure. (*a*) Overall fold of the LMO4 structure. The protein is depicted in cartoon representation and coloured grey. Residues with S atoms or that interact with zinc ions are shown as sticks, with sulfur, oxygen and nitrogen coloured green, red and blue, respectively. Zinc ions are represented by orange spheres. (*b*) Close-up view of zinc ion binding sites. Anomalous difference Fourier maps are represented as gold mesh and contoured at 4σ. Data sets collected above and below the Zn *K* edge are shown in the top and bottom rows, respectively. The numbers at the bottom right of each panel correspond to the anomalous peak heights (σ).

LMO4, a DNA-binding protein that contains four zinc ions (Fig. 2 ▸*a*; Deane *et al.*, 2004 ▸), was used to demonstrate the workflow for the identification of zinc ions by anomalous scattering. Two data sets were collected from a single LMO4 crystal; one above (λ = 1.2853 Å) and one below (λ = 1.3051 Å) the Zn *K* edge (Table 1 ▸; Figs. 1 ▸*a* and 1 ▸*b*). Anomalous difference Fourier maps were generated for both data sets, clearly showing the presence of four zinc metal ions in the data set collected above the Zn *K* edge (Fig. 2 ▸*b*). The anomalous peaks overlay with the previously modelled zinc ions. In the data set collected below the absorption edge, the theoretical zinc *f*′′ decreases to the level of sulfur (*f*′′ = 0.4 and 0.5 e^−^ for sulfur and zinc, respectively; Fig. 1 ▸), and both anomalous scatterers are visible in the anomalous difference Fourier maps. One would expect the zinc anomalous signal to vanish below the edge; however, due to the high data quality the anomalous signal from both sulfur and zinc can still be observed.

Furthermore, the zinc anomalous peak heights can also be directly evaluated from the peak-list file (.lsa) generated by *ANODE* (Thorn & Sheldrick, 2011 ▸; Table 3 ▸, Fig. 2 ▸*b*). For each zinc-binding site, the anomalous peak heights decrease threefold between the data sets collected above and below the Zn *K* edge, confirming the presence of zinc.

**Figure 3:**
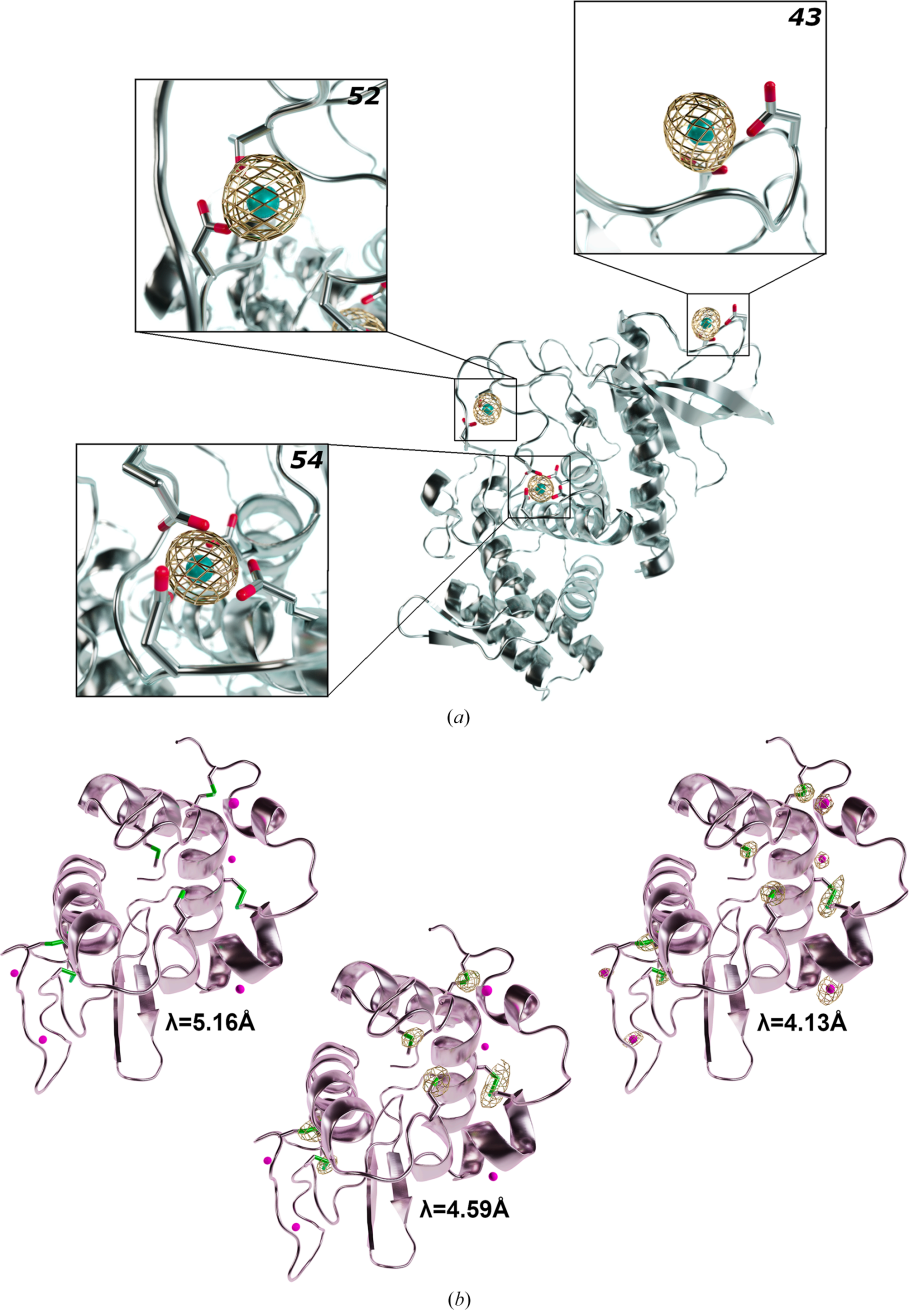
Calcium and chlorine identification in the thermolysin and lysozyme structures. (*a*) The thermolysin structure is depicted in cartoon representation and coloured grey. The three calcium ions are shown as cyan spheres. Aspartate and glutamate residues interacting with calcium ions are shown as sticks, with oxygen coloured red. The anomalous difference Fourier map corresponding to the data set collected above the calcium edge is displayed as gold mesh and contoured at 4σ. The numbers at the top right of each panel correspond to the anomalous peak heights (σ). (*b*) The lysozyme structure is depicted in cartoon representation and coloured light pink. Chlorine ions are shown as spheres and coloured magenta. Cysteine and methionine residues are shown as sticks, with S atoms coloured green. Anomalous difference Fourier maps are shown as gold mesh and contoured at 4σ. Left: at λ = 5.1660 Å, no anomalous peaks are observed around chlorine ions or S atoms from the data set collected below the Cl and S *K* edges. Middle: at λ = 4.5920 Å, the data set collected above the S *K* edge and below the Cl *K* edge shows only S atoms in anomalous difference Fourier maps. Right: at λ = 4.1328 Å, the data set collected above the S and Cl *K* edges shows both S atoms and chlorine ions.

The absorption edges of certain elements can only be exploited on synchrotron beamlines capable of accessing longer wavelengths (λ > 2 Å), such as beamline I23 at Diamond Light Source (Wagner *et al.*, 2016 ▸) and BL-1A at the Photon Factory (Liebschner *et al.*, 2016 ▸). The thermolysin crystal used in this study contains three calcium ions in addition to a zinc ion. The Ca *K* edge is located at λ = 3.0704 Å and is only within reach of long-wavelength beamlines. To identify and locate calcium ions, data sets were collected at two wavelengths: λ = 3.0689 Å (peak) and λ = 3.0804 Å (below the peak) (Fig. 1 ▸). These values are very close to each other and were selected based on the analysis of a calcium absorption-edge scan (Fig. 1 ▸*c*). Despite the small difference between the wavelengths (0.0115 Å), and like the zinc ions in LMO4, a drastic difference in the calcium anomalous signal was observed between wavelengths, confirming the presence of the three calcium ions in the structure (Fig. 3 ▸*a*). The anomalous peak heights for the three calcium ions range from 54σ to 43σ in the data set collected at the peak wavelength. Below the peak there is a large decrease in anomalous peak height, with values ranging between 7σ and 5σ (Table 3 ▸). Sigma (σ) refers to the standard deviation of the electron-density values in the Fourier anomalous difference map and is a measure of the anomalous signal compared with the noise.

Finally, even lighter elements, which show only very weak anomalous signal at the wavelengths typically used for macromolecular crystallography, can be identified, such as chlorine, even though the Cl *K* edge is at the very long wavelength of λ = 4.3929 Å. For demonstration, we collected three data sets from a laser-shaped lysozyme crystal: above and below the Cl and S *K* edges (λ = 4.1328 Å and λ = 5.1660 Å) and between them at λ = 4.5920 Å (Fig. 1 ▸). At λ = 4.1328 Å anomalous signal for both chlorine and sulfur can be observed, whereas at λ = 4.5920 Å only S atoms are detected and at λ = 5.1660 Å no anomalous signal is present for either chlorine or sulfur (Table 3 ▸). The superposition and comparison of anomalous difference Fourier maps clearly shows the locations of six chloride ions as well as all S atoms present in methionine and cysteine residues (Fig. 3 ▸*b*).

### 3.2. Element identification with *f*′′ refinement

The aim of the refinement procedure is to optimize the fit between the observed diffraction intensities and the calculated intensities derived from a structural model. Anomalous scattering effects, which can be significant for certain elements at certain X-ray wavelengths, can be included in refinement procedures to improve the accuracy of the resulting model. Refining *f*′′ involves adjusting its value for each type of atom in the crystal to minimize discrepancies between the observed and calculated diffraction data, particularly in regions where anomalous scattering effects are significant.

Refinement of *f*′′ can alternatively be used as a means to identify specific elements within a crystal structure. This is because, as stated earlier, the *f*′′ values are characteristic for each element and are known theoretically (Cromer & Liberman, 1970 ▸, 1981 ▸). By refining the *f*′′ values during the crystallographic refinement process and comparing them with the expected theoretical values for different elements, the presence of particular elements in the crystal can be deduced. This technique is particularly useful in cases where certain elements have distinctive *f*′′ values that can be differentiated from others. This technique is applicable for the identification of light elements in cases where access to absorption edges is limited.

Refinement of *f*′′ is not widely utilized, although it has previously been described and employed (Karasawa *et al.*, 2023 ▸; Liu *et al.*, 2013 ▸). To illustrate the procedure, *f*′′ was refined with *phenix.refine* (Liebschner *et al.*, 2019 ▸) for the three collected LMO4 data sets. These data sets were initially collected to perform a three-wavelength multiple anomalous dispersion (MAD) experiment at peak (λ = 1.2853 Å), inflection (λ = 1.2874 Å) and remote (λ = 1.3051 Å) wavelengths. Additionally, a zinc X-ray absorption-edge scan was measured to experimentally determine the *f*′′ values (Fig. 1 ▸*b*). As the data sets were collected near the Zn *K* edge, significant variations in *f*′′ were observed over a short wavelength range. Nevertheless, the *f*′′ refinement successfully identified these variations, yielding values closely matching the measured values (Table 4 ▸) and confirming the validity of this approach.

As mentioned earlier, calcium ions could be identified in the thermolysin structure with anomalous difference Fourier maps; however, this identification can also be performed with *f*′′ refinement with a single data set and for multiple ions. The thermolysin data set collected at λ = 3.0689 Å was also used for *f*′′ refinement for both zinc and calcium ions. The refined *f*′′ value for the single zinc ion was 1.5 e^−^ and the mean for the three calcium ions was 9.5 e^−^ (Table 4 ▸). These refined values closely align with the theoretical value of 2.3 e^−^ for zinc (Fig. 1 ▸*a*) and with the measured value of 8.5 e^−^ for calcium (Fig. 1 ▸*c*), effectively distinguishing calcium from zinc ions within the structure using a single data set.

Refinement of *f*′′ can also be employed in more complex scenarios, such as cases where the binding sites are not fully occupied. An example of this is observed in the potassium transporter NaK2K, where four potassium ions in the protein channel are situated on a fourfold crystallographic axis. Studies have indicated that the occupancy values for all of the potassium ions cluster around the maximum possible value of 0.25 (Langan *et al.*, 2018 ▸). This suggests that all four binding sites in the NaK2K selectivity filter are fully occupied with potassium ions rather than being co-occupied with water molecules. We have refined the *f*′′ values of these potassium ions using the determined occupancy of 0.25 (the ions are located on a fourfold symmetry axis), and the *f*′′ results corroborate the previously reported occupancy of 0.25, as the *f*′′ values for each potassium ion are similar to or higher than the theoretical value of 3.8 e^−^ at λ = 3.3500 Å (Table 4 ▸). If the binding sites were co-occupied by water molecules at 50% as postulated by the co-translation conduction mechanism, *f*′′ values that were halved or lower would be expected.

## 4. Conclusions

Elements can be identified through various methods, including electron-density difference maps, *B* factors, chemical environment, atom coordination or the *CheckMyMetal* server (Gucwa *et al.*, 2023 ▸). However, identification can become ambiguous, especially at lower resolutions where *B* factors are higher and bond distances are less accurate. Some experimental techniques, such as PIXE (Grime *et al.*, 2020 ▸), can identify the composition of elements in proteins but cannot pinpoint their locations. Anomalous scattering, which is specific to X-ray crystallography, is a preferred method for element identification and localization. Some programs, such as the ion-identification tool in *Phenix*, combine anomalous scattering with analysis of the chemical environment, occupancy and *B* factors (Echols *et al.*, 2014 ▸).

Element identification using anomalous difference Fourier maps is a powerful tool that does not necessarily require a fully refined structure for phase calculation; a partial model is often sufficient, although anomalous peak heights increase with a fully refined model. Since absorption edges are specific to chemical elements, it is possible to determine element identity and location by collecting two data sets: one above and one below the absorption edge. However, a drawback of this method is that a few important biological elements have absorption edges outside the range of standard synchrotron beamlines. While long-wavelength beamlines offer access to the absorption edges of elements such as calcium, potassium and chlorine, the absorption edges of sodium and magnesium are beyond reach. Although it is possible to measure anomalous signals from sodium (Karasawa *et al.*, 2023 ▸), collecting data below the Na *K* edge is not feasible. This experiment requires careful planning in advance, with specific wavelengths chosen for data collection and possibly an absorption-edge scan to determine the latter values. On the other hand, anomalous peaks are not dependent on the geometry of the binding site and are less influenced by the quality of the model, unlike *f*′′ refinement.

Refinement of *f*′′ requires an element to be modelled with the appropriate *B* factor and occupancy assigned; it is thus preferable to conduct *f*′′ refinement on a fully refined structure. The main advantage of *f*′′ is that it does not necessitate multiple data collections at specific wavelengths. However, if multiple elements are possible, the difference between their *f*′′ values at the recorded wavelength needs to be large enough to distinguish between them.

In summary, experimental data not only provide information to validate or correct AF2 model predictions, but can also contain anomalous data useful for element identification. At the very minimum, 360° of complete data with minimal radiation damage should be recorded to fully utilize the potential of anomalous scattering. Additionally, if possible, the wavelength should be chosen according to the desired experiment.

## Figures and Tables

**Table 1:** Data collection and processing Values in parentheses are for the outer shell.

	LMO4			Thermolysin		Lysozyme		
Diffraction source	I23, DLS			I23, DLS		I23, DLS		
Wavelength (Å)	1.2853	1.2874	1.3051	3.0685	3.0803	4.1328	4.5920	5.1660
Temperature (K)	80	80	80	80	80	80	80	80
Detector	PILATUS 12M	PILATUS 12M	PILATUS 12M	PILATUS 12M	PILATUS 12M	PILATUS 12M	PILATUS 12M	PILATUS 12M
Detector distance (mm)	250	250	250	250	250	250	250	250
Rotation range (°)	0.1	0.1	0.1	0.1	0.1	0.1	0.1	0.1
Total rotation range (°)	360	360	360	360	360	360	360	360
Exposure time (s)	0.1	0.1	0.1	0.1	0.1	0.1	0.1	0.1
Space group	*P*312	*P*312	*P*312	*P*6_1_22	*P*6_1_22	*P*4_3_2_1_2	*P*4_3_2_1_2	*P*4_3_2_1_2
*a*, *b*, *c* (Å)	61.8, 61.8, 93.4	61.8, 61.8, 93.4	61.8, 61.8, 93.4	93.2, 93.2, 129.0	93.2, 93.2, 129.0	79.0, 79.0, 36.9	79.0, 79.0, 36.9	79.0, 79.0, 36.9
α, β, γ (°)	90, 90, 120	90, 90, 120	90, 90, 120	90, 90, 120	90, 90, 120	90, 90, 90	90, 90, 90	90, 90, 90
Mosaicity (°)	0.33	0.33	0.34	0.22	0.21	0.07	0.07	0.07
Resolution range† (Å)	53.5–1.8 (1.83–1.80)	53.5–1.8 (1.83–1.80)	53.5–1.8 (1.83–1.80)	129.4–2.1 (2.14–2.10)	129.4–2.1 (2.14–2.10)	79.0–2.7 (2.75–2.70)	79.6–3.0 (3.05–3.00)	79.0–3.4 (3.43–3.37)
Total No. of reflections	362632	362489	361249	457328	454628	47562	34438	24281
No. of unique reflections	19257	19257	19257	20017	20029	2680	1980	1421
Completeness (%)	100 (100)	100 (100)	100 (100)	100 (99.7)	100 (99.7)	76.8 (60.4)	76.7 (51.6)	77.4 (65.4)
Multiplicity	18.8 (17.9)	18.8 (17.9)	18.8 (17.9)	22.8 (17.2)	22.7 (15.6)	17.7 (7.3)	17.4 (7.7)	17.1 (6.1)
〈*I*/σ(*I*)〉	18.4 (0.5)	19.6 (0.7)	21.1 (0.7)	19.3 (4.2)	20.8 (4.3)	62.0 (18.9)	44.4 (21.6)	36.1 (18.7)
CC_1/2_	1 (0.35)	1 (0.46)	1 (0.61)	0.99 (0.98)	0.99 (0.98)	0.99 (0.99)	0.99 (0.99)	0.99 (0.99)
*R*_r.i.m._	0.11 (3.08)	0.09 (2.45)	0.08 (1.91)	0.11 (0.27)	0.11 (0.28)	0.07 (0.12)	0.08 (0.082)	0.09 (0.072)
Wilson *B* factor (Å^2^)	28.5	28.7	28.5	25.2	26.0	19.4	19.8	20.7

† Maximum resolution was selected with an outer shell CC_1/2_ > 0.35 or was limited by the detector.

**Table 2:** Structure solution and refinement

	LMO4	Thermolysin	Lysozyme
Wavelength (Å)	1.2853	3.0685	4.1328
Resolution range (Å)	53.53–1.80	80.67–2.10	55.88–2.70
Completeness (%)	99.7	100	76.3
No. of reflections			
Working set	18231	18906	2660
Test set	967	1048	138
Final *R*_cryst_/*R*_free_	0.1980/0.2330	0.1810/0.2190	0.1876/0.2171
No. of non-H atoms	1414	2615	1026
R.m.s. deviations			
Bond lengths (Å)	0.008	0.003	0.007
Angles (°)	1.66	1.17	1.76
Average *B* factor (Å^2^)	32.6	25.2	19.4
Ramachandran statistics (%)			
Most favoured	100	95.5	96.9
Allowed	0	4.5	3.1
Clashscore	2.8	1.3	2.5
*MolProbity* score	1.18	1.16	1.44
PDB code	9f5b	9f56	9gcv

**Table 3:** Anomalous peak heights (σ) detected by *ANODE* from anomalous difference Fourier maps Only peaks above the default *ANODE* threshold (4σ) are shown.

Structure	LMO4		Thermolysin		Lysozyme					
Anomalous scatterer	Zn		Ca		Cl			S		
Wavelength (Å)	1.2853	1.3051	3.0685	3.0803	4.1328	4.5920	5.1660	4.1328	4.5920	5.1660
Peak heights (σ)	37.9	12.4	54.1	6.9	11.4	—	—	8.7	10.8	—
	36.8	10.8	51.8	5.9	6.9			8.4	10.7	
	21.7	8.1	43.3	5.1	6.8			8.1	9.5	
	18.4	7.5			6.1			7.9	9.0	
					5.5			7.6	9.0	
					4.3			7.0	9.0	
								6.8	7.8	

**Table 4:** Refined *f*′′ values for ions present in LMO4 (four zinc ions), thermolysin (one zinc and three calcium ions) and NaK2K (four potassium ions)

Structure	LMO4			Thermolysin		NaK2K
Anomalous scatterer	Zn			Zn	Ca	K
Wavelength (Å)	1.2853	1.2874	1.3051	3.0685	3.0685	3.3500
Measured (or theoretical) *f*′′ (e^−^)	5.2	1.0	0.5	2.3†	8.5	3.9†
Refined *f*′′ (e^−^)	5.3	0.9	0.7	1.5	9.9	4.9
	5.1	0.9	0.5		9.2	4.9
	4.5	1.1	0.8		9.5	5.1
	4.7	1.0	0.5			5.3

† Theoretical *f*′′ values.

## References

[bb1] Chukhutsina, V. U., Baxter, J. M., Fadini, A., Morgan, R. M., Pope, M. A., Maghlaoui, K., Orr, C. M., Wagner, A. & van Thor, J. J. (2022). *Nat. Commun.* **13**, 6420. 10.1038/s41467-022-34137-4 PMC9616832 36307413

[bb2] Cromer, D. T. & Liberman, D. (1970). *J. Chem. Phys.* **53**, 1891–1898.

[bb3] Cromer, D. T. & Liberman, D. A. (1981). *Acta Cryst.* A**37**, 267–268.

[bb4] Deane, J. E., Maher, M. J., Langley, D. B., Graham, S. C., Visvader, J. E., Guss, J. M. & Matthews, J. M. (2003). *Acta Cryst.* D**59**, 1484–1486. 10.1107/s0907444903011843 12876360

[bb5] Deane, J. E., Ryan, D. P., Sunde, M., Maher, M. J., Guss, J. M., Visvader, J. E. & Matthews, J. M. (2004). *EMBO J.* **23**, 3589–3598. 10.1038/sj.emboj.7600376 PMC517615 15343268

[bb6] Diamond, R. (1974). *J. Mol. Biol.* **82**, 371–391. 10.1016/0022-2836(74)90598-1 4856347

[bb7] Echols, N., Morshed, N., Afonine, P. V., McCoy, A. J., Miller, M. D., Read, R. J., Richardson, J. S., Terwilliger, T. C. & Adams, P. D. (2014). *Acta Cryst.* D**70**, 1104–1114. 10.1107/S1399004714001308 PMC3975891 24699654

[bb8] Einsle, O., Andrade, S. L., Dobbek, H., Meyer, J. & Rees, D. C. (2007). *J. Am. Chem. Soc.* **129**, 2210–2211. 10.1021/ja067562o PMC2527600 17269774

[bb9] El Omari, K., Duman, R., Mykhaylyk, V., Orr, C. M., Latimer-Smith, M., Winter, G., Grama, V., Qu, F., Bountra, K., Kwong, H. S., Romano, M., Reis, R. I., Vogeley, L., Vecchia, L., Owen, C. D., Wittmann, S., Renner, M., Senda, M., Matsugaki, N., Kawano, Y., Bowden, T. A., Moraes, I., Grimes, J. M., Mancini, E. J., Walsh, M. A., Guzzo, C. R., Owens, R. J., Jones, E. Y., Brown, D. G., Stuart, D. I., Beis, K. & Wagner, A. (2023). *Commun. Chem.* **6**, 219. 10.1038/s42004-023-01014-0 PMC10570326 37828292

[bb10] Evans, G. & Pettifer, R. F. (2001). *J. Appl. Cryst.* **34**, 82–86.

[bb11] Grime, G. W., Zeldin, O. B., Snell, M. E., Lowe, E. D., Hunt, J. F., Montelione, G. T., Tong, L., Snell, E. H. & Garman, E. F. (2020). *J. Am. Chem. Soc.* **142**, 185–197. 10.1021/jacs.9b09186 31794207

[bb12] Gucwa, M., Lenkiewicz, J., Zheng, H., Cymborowski, M., Cooper, D. R., Murzyn, K. & Minor, W. (2023). *Protein Sci.* **32**, e4525. 10.1002/pro.4525 PMC9794025 36464767

[bb13] Hekkelman, M. L., de Vries, I., Joosten, R. P. & Perrakis, A. (2023). *Nat. Methods*, **20**, 205–213. 10.1038/s41592-022-01685-y PMC9911346 36424442

[bb14] Herdman, M., von Kügelgen, A., Kureisaite-Ciziene, D., Duman, R., El Omari, K., Garman, E. F., Kjaer, A., Kolokouris, D., Löwe, J., Wagner, A., Stansfeld, P. J. & Bharat, T. A. M. (2022). *Structure*, **30**, 215–228. 10.1016/j.str.2021.10.012 PMC8828063 34800371

[bb15] Holden, H. M. & Matthews, B. W. (1988). *J. Biol. Chem.* **263**, 3256–3260. 10.2210/pdb3tmn/pdb 3343246

[bb16] Holton, J. M. (2007). *J. Synchrotron Rad.* **14**, 51–72. 10.1107/S0909049506048898 PMC2806430 17211072

[bb17] Jumper, J., Evans, R., Pritzel, A., Green, T., Figurnov, M., Ronneberger, O., Tunyasuvunakool, K., Bates, R., Žídek, A., Potapenko, A., Bridgland, A., Meyer, C., Kohl, S. A. A., Ballard, A. J., Cowie, A., Romera-Paredes, B., Nikolov, S., Jain, R., Adler, J., Back, T., Petersen, S., Reiman, D., Clancy, E., Zielinski, M., Steinegger, M., Pacholska, M., Berghammer, T., Bodenstein, S., Silver, D., Vinyals, O., Senior, A. W., Kavukcuoglu, K., Kohli, P. & Hassabis, D. (2021). *Nature*, **596**, 583–589. 10.1038/s41586-021-03819-2 PMC8371605 34265844

[bb18] Karasawa, A., Liu, H., Quick, M., Hendrickson, W. A. & Liu, Q. (2023). *Crystals*, **13**, 183.

[bb19] Kissel, L. & Pratt, R. H. (1990). *Acta Cryst.* A**46**, 170–175.

[bb20] Langan, P. S., Vandavasi, V. G., Weiss, K. L., Afonine, P. V., el Omari, K., Duman, R., Wagner, A. & Coates, L. (2018). *Nat. Commun.* **9**, 4540. 10.1038/s41467-018-06957-w PMC6208422 30382100

[bb21] Lennartz, F., Jeoung, J.-H., Ruenger, S., Dobbek, H. & Weiss, M. S. (2022). *Acta Cryst.* D**78**, 238–247. 10.1107/S2059798321013048 PMC8805299 35102889

[bb22] Liebschner, D., Afonine, P. V., Baker, M. L., Bunkóczi, G., Chen, V. B., Croll, T. I., Hintze, B., Hung, L.-W., Jain, S., McCoy, A. J., Moriarty, N. W., Oeffner, R. D., Poon, B. K., Prisant, M. G., Read, R. J., Richardson, J. S., Richardson, D. C., Sammito, M. D., Sobolev, O. V., Stockwell, D. H., Terwilliger, T. C., Urzhumtsev, A. G., Videau, L. L., Williams, C. J. & Adams, P. D. (2019). *Acta Cryst.* D**75**, 861–877. 10.1107/S2059798319011471 PMC6778852 31588918

[bb23] Liebschner, D., Yamada, Y., Matsugaki, N., Senda, M. & Senda, T. (2016). *Acta Cryst.* D**72**, 728–741. 10.1107/S2059798316005349 27303793

[bb24] Liu, Q., Dahmane, T., Zhang, Z., Assur, Z., Brasch, J., Shapiro, L., Mancia, F. & Hendrickson, W. A. (2012). *Science*, **336**, 1033–1037. 10.1126/science.1218753 PMC3769101 22628655

[bb25] Liu, Q. & Hendrickson, W. A. (2017). *Methods Mol. Biol.* **1607**, 377–399. 10.1007/978-1-4939-7000-1_16 PMC5541782 28573582

[bb26] Liu, Q., Liu, Q. & Hendrickson, W. A. (2013). *Acta Cryst.* D**69**, 1314–1332. 10.1107/S0907444913001479 PMC3689535 23793158

[bb27] Ma, S., Damfo, S., Bowler, M. W., Mykhaylyk, V. & Kozielski, F. (2024). *Acta Cryst.* D**80**, 451–463. 10.1107/S2059798324004480 PMC11154595 38841886

[bb28] McCoy, A. J., Grosse-Kunstleve, R. W., Adams, P. D., Winn, M. D., Storoni, L. C. & Read, R. J. (2007). *J. Appl. Cryst.* **40**, 658–674. 10.1107/S0021889807021206 PMC2483472 19461840

[bb29] Millán, C., Keegan, R. M., Pereira, J., Sammito, M. D., Simpkin, A. J., McCoy, A. J., Lupas, A. N., Hartmann, M. D., Rigden, D. J. & Read, R. J. (2021). *Proteins*, **89**, 1752–1769. 10.1002/prot.26214 PMC8881082 34387010

[bb30] Murshudov, G. N., Skubák, P., Lebedev, A. A., Pannu, N. S., Steiner, R. A., Nicholls, R. A., Winn, M. D., Long, F. & Vagin, A. A. (2011). *Acta Cryst.* D**67**, 355–367. 10.1107/S0907444911001314 PMC3069751 21460454

[bb31] Perrakis, A. & Sixma, T. K. (2021). *EMBO Rep.* **22**, e54046. 10.15252/embr.202154046 PMC8567224 34668287

[bb32] Rozov, A., Khusainov, I., El Omari, K., Duman, R., Mykhaylyk, V., Yusupov, M., Westhof, E., Wagner, A. & Yusupova, G. (2019). *Nat. Commun.* **10**, 2519. 10.1038/s41467-019-10409-4 PMC6555806 31175275

[bb33] Sheldrick, G. M. (2010). *Acta Cryst.* D**66**, 479–485. 10.1107/S0907444909038360 PMC2852312 20383001

[bb34] Spatzal, T., Schlesier, J., Burger, E. M., Sippel, D., Zhang, L., Andrade, S. L., Rees, D. C. & Einsle, O. (2016). *Nat. Commun.* **7**, 10902. 10.1038/ncomms10902 PMC4793075 26973151

[bb35] Terwilliger, T. C., Liebschner, D., Croll, T. I., Williams, C. J., McCoy, A. J., Poon, B. K., Afonine, P. V., Oeffner, R. D., Richardson, J. S., Read, R. J. & Adams, P. D. (2024). *Nat. Methods*, **21**, 110–116. 10.1038/s41592-023-02087-4 PMC10776388 38036854

[bb36] Thomson, J. J. (1906). *Conduction of Electricity through Gases*, 2nd ed. Cambridge University Press.

[bb37] Thorn, A. & Sheldrick, G. M. (2011). *J. Appl. Cryst.* **44**, 1285–1287. 10.1107/S0021889811041768 PMC3246834 22477786

[bb38] Thornton, J. M., Laskowski, R. A. & Borkakoti, N. (2021). *Nat. Med.* **27**, 1666–1669. 10.1038/s41591-021-01533-0 34642488

[bb39] Wagner, A., Duman, R., Henderson, K. & Mykhaylyk, V. (2016). *Acta Cryst.* D**72**, 430–439. 10.1107/S2059798316001078 PMC4784674 26960130

[bb40] Winter, G. (2010). *J. Appl. Cryst.* **43**, 186–190.

[bb41] Winter, G., Beilsten-Edmands, J., Devenish, N., Gerstel, M., Gildea, R. J., McDonagh, D., Pascal, E., Waterman, D. G., Williams, B. H. & Evans, G. (2022). *Protein Sci.* **31**, 232–250. 10.1002/pro.4224 PMC8740827 34747533

[bb42] wwPDB Consortium (2019). *Nucleic Acids Res.* **47**, D520–D528. 10.1093/nar/gky949 PMC6324056 30357364

